# Microbial infection and treatment strategies in cancer patients

**DOI:** 10.3389/fmicb.2025.1582382

**Published:** 2025-10-13

**Authors:** Kejing Zhu, Zhibo Yuan, Jingli Li, Ailing Fu

**Affiliations:** ^1^College of Pharmaceutical Sciences, Southwest University, Chongqing, China; ^2^Department of Pharmacy, Xiangyang Central Hospital, Affiliated Hospital of Hubei University of Arts and Science, Xiangyang, Hubei, China

**Keywords:** infection, anticancer, antibacterial, targets, drug development

## Abstract

The interplay between microbes and cancer has garnered significant attention in life sciences. Clinically, microbial infections in cancer patients are common complications and one of the major causes of mortality. Cancer patients often experience compromised immune defenses, and conventional therapies—including radiotherapy, chemotherapy, and invasive surgery—further diminish their resistance to pathogens. Emerging evidence indicates that intratumoral microbes and their interactions with the tumor microenvironment exacerbate cancer cell proliferation, drug resistance, metastasis, and poor prognosis. However, complex multidrug regimens increase patient burden and reduce compliance. This necessitates the development of single agents with dual anticancer and antimicrobial properties. Promisingly, naturally derived compounds and synthetic chemicals exhibit such dual functionalities. This review introduces microbial contributions to oncogenesis and analyzes molecular targets of dual-function agents, proposing their potential as novel therapeutics to improve clinical outcomes.

## 1 Background

Microbial infections constitute a critical complication and leading mortality cause in cancer patients (Sepich-Poore et al., 2021; Helmink et al., 2019). Clinically, specific microbes correlate with malignant tumor behaviors, such as metastasis and drug resistance, and are linked to poor prognosis (Pang et al., 2024; Er et al., 2025). These microbes originate from both environmental sources and endogenous opportunistic pathogens. Their symbiotic relationship with tumor cells weakens immune defenses, exacerbating disease severity and mortality (Jiang et al., 2024; Shen et al., 2024; de Barros et al., 2024). However, the combination application of antibiotics and anticancer drugs currently faces thorny challenges, including dosing complexity, combination toxicity, drug interactions, and reduced compliance (Kubecek et al., 2021; Royo-Cebrecos et al., 2024).

Development of single agents with dual functions of anticancer and antibacterial activities has become an inevitable solution to address cancer complicated with bacterial infection. The agents not only inhibit the proliferation of cancer cells, but also eliminate bacteria at the same concentration, thereby block cancer and infection synergistic control, and simplify the medication regimens (Rai et al., 2024; Xie et al., 2024; Kumari et al., 2024). Preclinical studies demonstrate that such agents achieve long-term relapse-free survival (>700 days) and even complete remission (Wang et al., 2024). This review synthesizes current knowledge on microbial-tumor interactions and highlights advances in dual-functional drug development. This review introduces current knowledge on microbial-tumor interactions, and highlights the advances in the dual-functional drugs at the first time, which would provide a new insight into the development of this new category of drugs.

## 2 Infection in cancer patients

Immunocompromised cancer patients are highly susceptible to infections. Tumor cells play a pivotal role in undermining immune function. They secrete various immunosuppressive factors, such as transforming growth factor-β(TGF-β), interleukin-10 (IL-10), and prostaglandin E2 (PGE2) (Nduom et al., 2015; Motz and Coukos, 2013; Yaguchi and Kawakami, 2016). As the immune system weakens, the body becomes more susceptible to infections, and the presence of infections could, in turn, exacerbate the stress on the already compromised immune system of cancer patients (Kang et al., 2024). Cancer-associated immune dysfunction, compounded by cytotoxic therapies (chemotherapy, radiation, surgery, transplantation, and corticosteroids), creates a permissive environment for infections (Bastien et al., 2019).

Epidemiological analyses reveal ~20% of cancer patients develop sepsis, with infection-related mortality reaching 40–70% in immunocompromised cohorts (Boucher and Carpenter, 2020; Hensley et al., 2019; Nelmes et al., 2023). Diagnosing infections is challenging due to overlapping symptoms with cancer progression, such as low-grade fever, fatigue, and malaise (Yaegashi et al., 2014). Common pathogens include bacteria (*Staphylococcus, Streptococcus, Enterococcus, Pseudomonas aeruginosa, Klebsiella pneumoniae*) and fungi (*Candida, Aspergillus, Mucormycosis, Cryptococcus, Pneumocystis*) (Fentie et al., 2018; Petrikkos et al., 2024; Delgado and Guddati, 2021; Danielsen et al., 2023). To make matters worse, the increasing prevalence of antibiotic-resistant strains has severely complicated treatment, leaving these already vulnerable patients with limited effective treatment options.

## 3 Intratumoral microbe

Emerging evidence suggests that symbiotic bacteria and fungi reside within malignant tumor cells, as revealed by metagenomics and next-generation sequencing (Gao et al., 2022; Galeano Niño et al., 2022). A study analyzing fungal communities in over 17,000 tissue and blood samples from 35 cancer types detected microbial presence in all samples (Dohlman et al., 2022). Specific microbial communities are associated with gastrointestinal tumors, breast cancer, melanoma, lung cancer, bladder cancer, lymphomas, adenomas, and head and neck paragangliomas (Kim et al., 2024). For instance, *Fusobacterium* dominates colorectal cancer (CRC) niches, while *Malassezia* enriches in pancreatic tumors (Zepeda-Rivera et al., 2024; Aykut et al., 2019). Notably, tumor-resident microbes significantly impact therapeutic outcomes—for instance, intratumoral *Enterococcus* compromises Programmed Death-1 (PD-1) inhibitor efficacy in melanoma models (Matson et al., 2018). Mechanistically, intratumoral microbiota modulate tumor growth, metastasis, and treatment resistance through immune modulation, inflammation induction, and so on (Li and Saxena, 2022; Shi et al., 2024). These findings highlight the critical need for integrating targeted antimicrobial strategies into cancer therapeutic regimens to address microbial contributions to tumor progression and treatment failure.

## 4 Carcinogenic mechanisms of microbiota

Unlike normal cells, tumor cells could coexist with specific bacteria and fungi, forming a symbiotic microbiota that benefits both tumor and microbial proliferation and drug resistance (Lutsiv et al., 2024). For instance, fungi associated with lung cancer include *Aspergillus, Blastomyces, Cryptococcus, Malassezia, Candida, Bacillus*, while bacteria such as *Staphylococcus, Streptococcus, Pseudomonas aeruginosa (P. aeruginosa), Klebsiella, Escherichia coli (E. coli)* contribute to a cancer-promoting microenvironment. The presence of these microbes is strongly associated with elevated mortality rates and unfavorable prognoses in patients.

**Figure 1:**
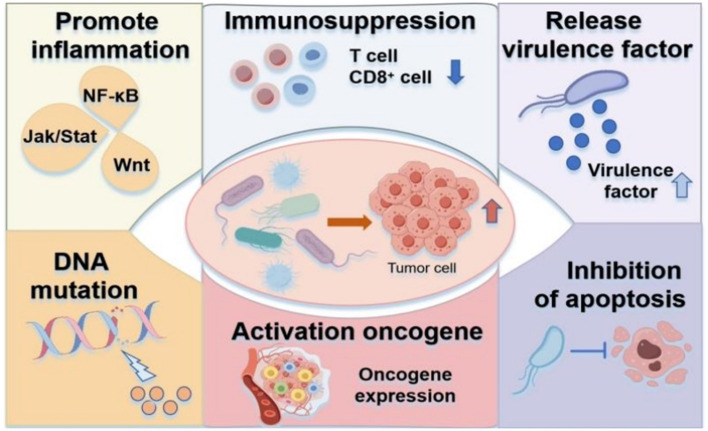
Carcinogenic mechanisms of microbiota. (1) Chronic inflammation from excessive immune activation. (2) Immune evasion via immunosuppressive mechanisms. (3) Virulence factor secretion. (4) DNA damage through mutagenic metabolites. (5) Oncogenic pathway activation. (6) Apoptosis resistance in tumor cells.

The carcinogenic effects of bacteria and fungi are mediated through several mechanisms (Figure 1): (1) Chronic inflammation induction; (2) Immunosuppression and immune evasion; (3) Secretion of virulence factors that act as carcinogens; (4) Induction of DNA mutations; (5) Activation of oncogene expression and signaling pathways; (6) Inhibition of tumor cell apoptosis.

### 4.1 Immunosuppression and inflammatory activation of *Porphyromonas gingivalis* (*P. gingivalis*)

*P. gingivali*s infection enhances programmed death-ligand 1 (PD-L1) expression on dendritic cells within tumor microenvironments and lymph nodes through Akt/STAT3 pathway activation, subsequently impairing CD8^+^ T cell functionality (Ren et al., 2023). This manifests as significant downregulation of interleukin-2 (IL-2), interferon-γ (IFN-γ), perforin, granzyme B, and CD107a expression in CD8^+^ T cells. In murine models, *P. gingivali*s-induced immunosuppression significantly enlarges tumor areas and accelerates oral cancer progression (Luo et al., 2024). Given its prevalence in human gingival microbiota, targeting *P. gingivalis* proliferation and its molecular signaling pathways could enhance CD8^+^ T cell activity and improve PD-1/PD-L1 immunotherapy efficacy.

Furthermore, *P. gingivalis* promotes malignancy in gastrointestinal cancers, including CRC and esophageal cancer. CRC mouse models administered *P. gingivalis* exhibit increased colorectal tumor burden compared to controls (Wang et al., 2021). Mechanistically, *P. gingivalis* recruits myeloid immune cells to activate the NLRP3 inflammasome, driving excessive inflammation that facilitates CRC initiation and progression.

### 4.2 Induction of chronic inflammation by *Candida albicans* (*C. albicans*)

*C. albicans* promotes carcinogenesis through chronic inflammation by orchestrating a cascade of molecular and immunological disruptions. Studies have demonstrated that *C. albicans* is closely associated with oral malignancies. Recent research has elucidated several mechanisms underlying this association, including disruption of the epithelial barrier, production of carcinogenic substances (e.g., nitrosamines and acetaldehyde), and induction of chronic inflammation (Liao et al., 2020). Concurrently, *C. albicans* disrupts mucosal barriers via hydrolases (e.g., proteases), enabling persistent microbial invasion and chronic antigen exposure. This triggers sustained activation of NF-κB and STAT3 pathways, driving the production of proinflammatory cytokines (IL-6, TNF-α, and IL-1β) that fuel hyperproliferation and apoptosis resistance in epithelial tissues (Wang X. et al., 2023).

The interplay between dysregulated immune responses and fungal persistence further exacerbates oncogenic processes. In genetically susceptible hosts, such as those with chronic mucocutaneous candidiasis, defective IFN pathways amplify inflammatory damage and epithelial dysplasia (Smeekens et al., 2013). Additionally, *C. albicans* synergizes with bacterial pathogens (e.g., *Streptococcus*) to activate TLR2/4 and NLRP3 inflammasomes, amplifying IL-1β-driven inflammation and reactive oxygen species (ROS) generation, both implicated in malignant transformation (Wang X. et al., 2023). Animal models of oral carcinogenesis confirm these mechanisms, where antifungal therapy reduces squamous hyperplasia and carcinoma incidence, directly linking *C. albicans* to inflammation-mediated oncogenesis (Sano et al., 2009).

### 4.3 Promotion of tumor cell immune evasion by *Malassezia*

*Malassezia*, an opportunistic pathogenic fungus commensal on human skin, exhibits conditional pathogenicity contingent upon host immune status. Emerging evidence indicates that immunosuppression facilitates fungal translocation into internal organs, thereby potentiating tumorigenesis and malignant progression (Spatz and Richard, 2020). Notably, *Malassezia* demonstrates tissue tropism for multiple malignancies, including pancreatic ductal adenocarcinoma (PDA), breast cancer, gastric cancer, and prostate cancer. Quantitative analysis reveals a remarkable 3,000-fold enrichment of fungal biomass in PDA tumor tissues compared to adjacent normal parenchyma, a phenomenon conserved across human and murine model (Aykut et al., 2019). In breast cancer patients, fungal detection in tumor specimens correlates with significantly reduced overall survival rates compared to *Malassezia*-negative cohorts (Liu et al., 2024).

The mechanistic underpinnings involve immunomodulatory pathways mediated by *Malassezia*. Notably, gastric cancer specimens with fungal colonization exhibit concomitant upregulation of PD-L1 expression, suggesting fungal-mediated immune evasion through checkpoint protein induction (Zhang et al., 2022). This immunoregulatory axis may explain the observed acceleration of tumor progression in colonized malignancies.

### 4.4 Release of virulence factors from *Helicobacter pylori* (*H. pylori*)

*H. pylori* is a well-established driver of gastric carcinogenesis, implicated in ~90% of gastric cancer cases (Engelsberger et al., 2024). It also associates with malignancies such as colon cancer, pancreatic cancer, and gastric mucosa-associated lymphoid tissue lymphoma. Notably, 80% of infected individuals remain asymptomatic despite severe potential consequences.

The procarcinogenic activity of *H. pylori* primarily stems from virulence factor secretion. Cytotoxin-associated gene A (CagA), a key virulence factor, undergoes phosphorylation by host c-Src/c-Abl kinases, activating oncogenic signaling pathways that induce gastric epithelial inflammation, precancerous lesions, and adenocarcinoma (Zhao et al., 2024). Additional virulence factors include vacuolating cytotoxin A (VacA), which triggers immune cell apoptosis, and γ-glutamyl transpeptidase (γ-GT), which disrupts gastric epithelial integrity and promotes immune tolerance (Wang B. et al., 2023; Baskerville et al., 2023). Targeting *H. pylori* infection may thus complement existing cancer therapies.

### 4.5 Induction of tumor cell DNA mutation by polyketide-non-ribosomal peptide synthase operon (pks^+^) *E. coli*

The genotoxic pks^+^ *E. coli* contributes to colorectal carcinogenesis through a mutagenic mechanism mediated by its secondary metabolite, colibactin (Pleguezuelos-Manzano et al., 2020). Pks^+^ *E. coli* produces colibactin, a secondary metabolite synthesized by the pks genomic island. Colibactin acts as a crosslinking agent, forming covalent interstrand crosslinks between adenine residues in double-stranded DNA. Colibactin induces DNA interstrand crosslinks, primarily targeting adenine residues, which obstruct replication forks and lead to double-strand breaks during S phase. This damage triggers error-prone repair processes, such as non-homologous end joining, resulting in characteristic mutational signatures. These mutations preferentially affect cancer driver genes (e.g., APC and KRAS) and promote chromosomal instability through chromothripsis-like rearrangements. Clinically, pks^+^ *E. coli* colonization correlates with elevated mutation burden and adverse prognosis, highlighting its role as a microbial driver of oncogenic mutagenesis in colorectal cancer.

### 4.6 Activation of oncogenic signal pathways by *Fusobacterium nucleatum* (*F. nucleatum*)

Intestinal microbiota such as *F. nucleatum is* strongly linked to CRC pathogenesis (Garrett, 2019). *F. nucleatum* exerts potent procancer effects *in vitro* and *in vivo*, notably enhancing CRC tumor growth in murine models. Its primary carcinogenic mechanism involves FadA adhesion protein-mediated activation of the Wnt/β-catenin pathway via E-cadherin binding (Cohn et al., 2023; Rubinstein et al., 2013). Additionally, *F. nucleatum* increases proliferation of colorectal cancer cells and tumor development in mice by activating toll-like receptor 4 signaling (TLR4) to nuclear factor -κB (NF-κB), and up-regulating expression of microRNA-21 (Yang et al., 2017). Moreover, *F. nucleatum* colonization accelerates breast cancer metastasis, highlighting its systemic oncogenic potential (Fu et al., 2022).

## 5 Progress of single agents for anticancer and antibacterial activities

The interdependent relationship between tumor cells and microbes presents a formidable obstacle, making it challenging to entirely halt tumor growth and recurrence through the exclusive use of anticancer treatments or antibacterial medications (Kapoor et al., 2022; Ma et al., 2023). In clinical settings, established anticancer interventions, such as chemotherapy, radiotherapy, and invasive surgical methods, typically succeed in restraining tumor cell proliferation. However, their effectiveness against the bacteria and fungi associated with tumors remains notably inadequate (Inamura, 2023; Sheng et al., 2024). Given these difficulties, single agents exhibiting broad-spectrum antimicrobial and anticancer capabilities are surfacing as promising prospects for both preclinical and clinical exploration (Rohilla et al., 2014; Yadav et al., 2023).

### 5.1 Overall mechanism of single agents with anticancer and antimicrobial effects

Natural products, clinically established antimicrobial and antitumor agents, and newly synthesized single compounds have been identified as pivotal sources for developing novel antimicrobial and antitumor therapeutics. Diverse phytochemicals, such asalkaloids, sesquiterpenes, flavonoids, and saponins, demonstrate broad-spectrum bioactivities encompassing antioxidant, anti-inflammatory, antimicrobial, and antitumor properties. Furthermore, clinically validated dual-activity drugs and novel synthetic bifunctional compounds exhibit multimodal biological effects, including antibacterial, antifungal, and antitumor activities. These agents execute their pharmacological actions via diverse mechanisms of action (Table 1), ranging from microbial membrane disruption to tumor cell apoptosis induction. These findings underscore the concurrent antimicrobial and antitumor capacities inherent in both natural and synthetic compounds, highlighting substantial potential for designing multifunctional therapeutic agents targeting the concurrent management of infectious diseases and malignancies.

**Table 1:** Main mechanisms of the typical dual-functional agents for anticancer and antibacteria.

**Agents**	**Anticancer mechanism**	**Antibacterial mechanism**	**Reference**
**Naturally-originating chemicals**			
Rapamycin	Inhibiting mammalian target of rapamycin (mTOR) signaling pathway	Inhibiting mTOR signaling pathway	Bian et al., 2020; Hujber et al., 2017; Jiang et al., 2021
Curcumin	Cell cycle arrest; apoptosis activiation; inhibiting Wnt/β-catenin signal pathway	Disrupting cell membrane permeability	Patel et al., 2020; Wong et al., 2021; Dong et al., 2022; Vallee et al., 2019
Berberine	Cell cycle arrest; apoptosis activiation; inhibiting Hedgehog signal pathway	Binding with bacterial DNA and interfering protein synthesis	Xiong et al., 2022; Wang et al., 2015; Cao et al., 2016
xanthyletin	Cell cycle arrest; inducing cell apoptosis; anti-angiogenesis	Destroying cell walls and membranes of bacteria	Porrello et al., 2025; Zhang et al., 2014
Allicin	Cell cycle arrest; inducing cell apoptosis; increasing ROS level; activating immune defense system	Destroying cell membranes of bacteria	Tsubura et al., 2011; Tao et al., 2023
**Antimicrobial peptides**			
LL-37	Downregulating the expression of vascular endothelial growth factor (VEGF); inhibiting tumor angiogenesis; inducing cell apoptosis	Destroying cell membranes of bacteria	Nagaoka et al., 2020; Bhattacharjya et al., 2024; Stakheev et al., 2022
Human β-defensin	Cell cycle arrest; inducing cell apoptosis; enhancing immune surveillance ability;	Destroying cell membranes of bacteria	Hanaoka et al., 2016; Lee et al., 2013; Adyns et al., 2023
Buforin II	Targeting mitochondria and interfering respiratory chain function and ATP synthesis; inducing cell apoptosis	Destroying cell membranes of bacteria; binding to DNA and interfering DNA replication	Lee et al., 2008; Perez et al., 2019
Peptide-P2	Interfering mitochondrial function; activating cell apoptotic signaling pathways in tumor cells	Destroying cell membranes of bacteria; Interacting with nucleic acids and inhibiting DNA replication	Xu et al., 2018; Zhang Q. et al., 2021
**Conventional drugs in new function**			
Macrolide clarithromycin	Blocking potassium ion channels, and affecting the electrophysiological balance of tumor cells	Binding with the P site of 50S subunit of bacterial ribosomes, and blocking protein synthesis	Takeda et al., 2020; Bui et al., 2025
Thiabendazole	Targeting chromosome maintenance protein 2 and blocking the initiation stage of DNA replication; inhibiting tumor angiogenesis	Inhibiting microtubule protein polymerization required for cell division and interfering the process of mitosis	Yadav et al., 2016; Bai et al., 2024
Natamycin	Activating oxidative stress-induced cell apoptosis pathway	Binding with ergosterol in fungal cell membranes, and increasing membrane permeability	An et al., 2021; Kang et al., 2025
Itraconazole	Blockade of Hedgehog and HER2/AKT signaling pathway	Inhibiting fungal cytochrome P-450 dependent enzymes, and blocking ergosterol biosynthesis, leading to membrane integrity damage and fungal death	Zhang W. et al., 2021; Kulkarni et al., 2025; Olender et al., 2025
Osimertinib	Third-generation EGFR inhibitor	Inhibiting the endocytosis of C. albicans into host cells	Liu et al., 2022; Singh et al., 2025
**Other dual-functional therapies**			
Minor groove binders	Inhibiting replication or transcription	Interfering with DNA unwinding and nuclease functioning	Msallam et al., 2025; Alniss et al., 2024
Photodynamic therapy	Increasing ROS level	Increasing ROS level	Chen et al., 2024; Li et al., 2025
Au@Ag2Se-FA	Increasing immune surveillance and defense ability	Disrupting the cellular structures and functions	Wang et al., 2024
Ph-ph^+^	Disrupting mitochondrial function	Reducing energy supply	Zhao et al., 2022a,b
Quinoline-5-sulfonamides	Inhibiting NF-κB signal pathway; inducing cell apoptosis	Interfering DNA replication; Inhibiting topoisomerase	Zieba et al., 2024; Qurban et al., 2024

**Figure 2:**
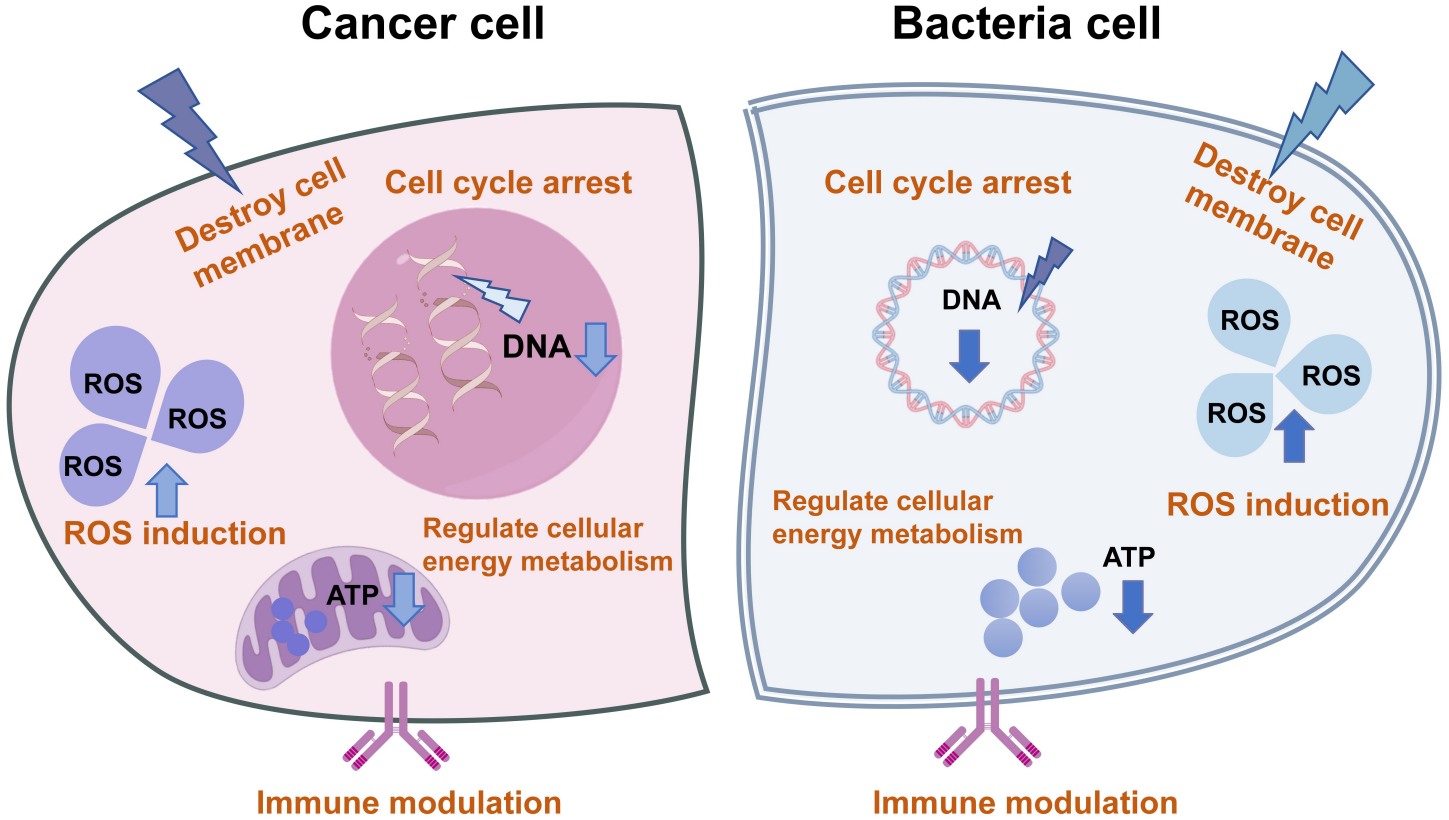
The molecular mechanism of single agents against cancer and microbia. These agents typically exert dual inhibitory effects on both cancer and bacterial pathogens through multifaceted mechanisms, including cell cycle arrest, modulation of energy metabolism pathways, immunoregulatory functions, enhancement of membrane permeability, and induction of ROS generation.

Dual-functional agents eliminate microbes and cancer cells through multiple mechanisms (Figure 2): (1) Cell cycle arrest: Targeting the uncontrolled proliferation shared by microbes and tumor cells. (2) Regulation of cell energy metabolism. (3)Immune modulation: Enhancing immune surveillance and defense capabilities. (4) Membrane permeabilization: Increasing cell membrane permeability to induce cytotoxicity. (5) ROS induction: Promoting localized ROS overproduction to eradicate pathogens and tumor cells.

### 5.2 Naturally-originating single compounds with antibacterial and anticancer effects

Compounds with anticancer and antimicrobial properties, such as alkaloids, sesquiterpenes, flavonoids, and saponins, are widely distributed in nature. Natural products and their derivatives with antibacterial and anticancer activities, including rapamycin, curcumin, berberine, xanthoxylin, allicin, quercetin, solanine, quinalizarin, and evodiamine, have been intensively investigated (Fan and Yao, 2022; Vuong, 2021). These natural compounds have shown remarkable potential in both combating cancer cells and inhibiting the growth of various microbes. For instance, rapamycin, initially discovered as an antifungal agent, has now been extensively studied for its anti-cancer properties (Ganesh and Subathra, 2023). It functions by inhibiting the mammalian target of rapamycin (mTOR) pathway, which plays a crucial role in cell growth, proliferation, and survival, thus impeding the development of tumors. Curcumin, a major component of turmeric, has potent antioxidant and anti-inflammatory properties (Abd El-Hack et al., 2021). In cancer treatment, it could induce apoptosis in cancer cells, modulate multiple signaling pathways, and also exhibits antibacterial activity against a wide range of bacteria, including both Gram-positive and Gram-negative strains (Weng and Goel, 2022). Berberine, found in plants like *Coptis chinensis*, has been shown to have antibacterial effects by intBerberineerfering with bacterial DNA replication and cell membrane integrity (Khan et al., 2022). In the context of cancer, it could suppress tumor cell growth, invasion, and metastasis through various mechanisms such as regulating gene expression and cell cycle progression (Xiong et al., 2022).

Antimicrobial peptides (AMPs), such as LL-37, Human β-defensin, Buforin II, Peptide-P2, demonstrate dual antimicrobial and antitumor activities through diverse mechanisms (Wu et al., 2021; Baindara et al., 2018; Min et al., 2024; Girdhar et al., 2024). Membrane disruption is a common antibacterial strategy: cationic AMPs bind to negatively charged bacterial membranes via electrostatic interactions, forming pores or dissolving lipid bilayers, leading to cell lysis (Pérez-Peinado et al., 2018; Dong et al., 2014). Similarly, lactoferrin-derived peptides disrupts microbial membranes while modulating immune responses to enhance pathogen clearance (Gruden and Poklar, 2021).

In antitumor activity, AMPs exploit cancer cells' altered membrane properties (e.g., phosphatidylserine exposure). Melectin penetrates cancer cell membranes and binds intracellular DNA or RNA, inhibiting replication and inducing apoptosis (Liang et al., 2021). Defensins may trigger mitochondrial dysfunction or activate death receptors in tumors, akin to Bogorol B-JX's ROS-mediated apoptosis (Jiang et al., 2017). These mechanisms, combined with low resistance development, underscore AMPs as versatile therapeutic candidates.

### 5.3 New function of clinical antibiotics and anticancer drugs

Dactinomycin, also known as actinomycin D, is an antibiotic isolated from the bacterium Streptomyces parvulus. It has long been recognized for its potent antibacterial properties, but in recent years, its anticancer activity has attracted significant attention. It is a key component in the treatment of Wilms' tumor, a common kidney cancer in children (Green et al., 1993; Armstrong et al., 2025). It is also used in the treatment of rhabdomyosarcoma, a type of soft-tissue sarcoma, and orther soft tissue sarcomas (Koscielniak et al., 2024). The primary mechanism by which dactinomycin exerts its anticancer effects is through its ability to intercalate into DNA. It specifically binds to the guanine-cytosine (G-C) base pairs in the DNA double helix. This binding disrupts the normal function of DNA-dependent RNA polymerase, thereby inhibiting transcription (Passaquin et al., 1998). Moreover, dactinomycin could also induce DNA strand breaks, leading to genomic instability and ultimately triggering apoptosis in cancer cells.

Furthermore, antifungal agents have also been found to possess anticancer activity. Thiabendazole (TBZ) is a widely used generic antifungal drug with over 50 years of clinical application and high safety. Its antifungal mechanism lies in inhibiting the formation of microtubules during fungal mitosis. Later, it was serendipitously discovered that TBZ has potential anticancer activity (Garge et al., 2021; Hu et al., 2022). In a mouse model of fibrosarcoma (a connective-tissue tumor with a rich vascular network), TBZ reduces angiogenesis in the tumor by more than 50%, thereby delaying tumor growth and metastasis. The vascular damage caused by TBZ is related to a decrease in microtubule protein, which is similar to its antifungal mechanism.

Natamycin (NAT) is a polyene zwitterionic macrolide antibiotic that exerts antifungal effects by binding to ergosterol on fungal cell membranes. Recently, An et al. reported that NAT could induce liver cancer cell apoptosis both *in vitro* and *in vivo*. The mechanism is closely associated with the down-regulation of the expression of peroxisome protein 1, which leads to a significant increase in ROS accumulation (An et al., 2021). In addition, the blockade of the AKT/mTOR signal pathway is also involved in the anticancer activity of NAT. This blockade induces cell-protective autophagy and reduces the acquired drug resistance of cancer cells.

Itraconazole is a commonly used antifungal medication in clinical. It is found that has the ability to significantly increase the overall survival rate of tumor-bearing animalst as combination with antitumor drugst (Kast, 2024). In clinical trials (NCT02749513), itraconazole is orally administered to patients with esophageal cancer, including adenocarcinoma (EAC) and squamous cell carcinoma (ESCC), and the results show that itraconazole has potent antitumor properties, partially through blockade of HER2/AKT signaling pathway (Zhang W. et al., 2021). Moreover, another antifungal agent, clotrimazole, can inhibit cancer cell division and proliferation by blocking the entry of calcium and potassium ions into the cells (Cortat and Zobi, 2023).

The third-generation EGFR inhibitor osimertinib (OSI) has a dual function. In addition to being an anticancer drug, it can also be used in the treatment of fungal infections. OSI could target the host EGFR receptor to inhibit the endocytosis of Candida albicans into host cells, thereby protecting the host cells from fungal invasion (Liu et al., 2022). Additionally, OSI could bind to the drug efflux pump Pdr5 of *C. albicans* and suppress the efflux of the antifungal drug fluconazole (FLC). This action increases the accumulation of FLC in cells and improves the antifungal efficacy of FLC. Thus, OSI combined with FLC has broad-spectrum antifungal efficacy against multiple fungal resistant strains.

Certain antibiotics, such as thiabendazole, as well as anticancer drugs like actinomycin D, exhibit dual functionalities of antibacterial and anticancer activities, enabling the simultaneous treatment of infections and cancer. These medications have been extensively applied in clinical practice, with their safety and efficacy well-established. Their mechanisms of these drugs, which include inhibiting microtubule formation or inducing DNA strand breaks, have been intensively investigated. However, the relatively low antibacterial or anticancer activity of these drugs restricts their widespread application. Moreover, some drugs exhibit a narrow therapeutic window, manifested as dose-limiting toxicity.

### 5.4 Development of single compounds with antibacterial and anticancer activities

Based on the interplay between tumor cells and microbial infections, diverse single compounds exhibiting both antimicrobial and anticancer properties have been synthesized. The mechanisms of action of these drugs include inhibiting the cell cycle, inducing the generation of ROS, regulating the immune system, and inhibiting energy metabolism, etc.

#### 5.4.1 Inhibition of cell cycle

Minor groove binders (MGBs) exhibit antitumor and antibacterial properties. These agents generally induce either permanent DNA damage or temporary inactivation of DNA (Msallam et al., 2025). Upon binding, MGBs disrupt interactions between critical proteins (e.g., transcription factors and polymerases) and DNA in tumor cells through allosteric perturbation, thereby inhibiting replication or transcription processes (Alniss et al., 2024). Notably, MGBs such as MGB28 and MGB32 demonstrate potent inhibitory effects against Gram-positive bacteria and Gram-negative bacteria. Their antibacterial mechanism involves binding to the minor groove of bacterial DNA, which interferes with DNA unwinding or impairs nuclease functionality.

#### 5.4.2 Induction of ROS generation

In recent years, several biomaterials have been synthesized that exhibit both anticancer and antibacterial activities. The underlying mechanism of these biomaterials against tumors and bacteria primarily involves increasing intracellular ROS levels. This leads to irreparable oxidative damage to bacterial cell membranes and other functional biomolecules such as DNA, enzymes, and fatty acids, thereby exerting cytotoxic effects on both bacteria and tumor cells. These bifunctional nanomaterials encompass inorganic nanomaterials, organic polymer materials, and photosensitizers. Notably, inorganic nano antibacterial agents are a type of anticancer and antibacterial material that capitalizes on the properties of inorganic metal ions (Shi et al., 2018; Tehrani Nejad et al., 2022). Once these nanomaterials enter cells, they could promote bacteria to generate ROS, causing cell deformation, collapse, and ultimately cell death (Jing et al., 2024).

Photodynamic therapy (PDT) is an outstanding technique for treating tumors and microbial infections. It utilizes photosensitive agents and laser activation to generate ROS (Xu et al., 2024; Law et al., 2024). This technique works by injecting a photosensitizer into animals and then irradiating the lesion site with a specific wavelength. This process facilitates the selective accumulation of the photosensitizer in the lesion, triggering a photochemical reaction to generate excessive ROS and destroy tumors and microbes (Zhang J. et al., 2024). Notably, aggregation-induced emission (AIE) photosensitizers in the PDT technique have been extensively employed in photodynamic anticancer and antibacterial therapy (Xu et al., 2025). Among the reported organic photosensitizers, AIE photosensitizers have unique aggregation-induced enhancement effects on ROS production. They have been successfully applied in microbial and tumor detection, identification, and treatment (Yang et al., 2021).

#### 5.4.3 Improvement of immune surveillance and defense ability

Tumor resident intracellular microbiota (TRIM) has recently garnered significant attention as a crucial factor in carcinogenic processes (Fu et al., 2022; Ma et al., 2021). The immunosuppressive tumor microenvironment (TME) serves as a haven for TRIM. Accumulating evidence indicates that TRIM can not only reduce the efficacy of chemotherapy but also enhance drug toxicity to normal tissues, while simultaneously promoting the pro-inflammatory response and proliferation of tumor cells (Geller et al., 2017; Sears et al., 2014).

In this regard, Yuanlin Wan et al. proposed a solution to the issues of immunosuppression and over-growth in TRIM-infected cancers by incorporating TRIM-targeted antimicrobials into anti-tumor therapy (Wang et al., 2024). To enable simultaneous antimicrobial and anti-tumor therapy with a single drug, they designed Ag_2_Se shell-coated Au nanoparticles whose surface was modified with folic acid (Au@Ag_2_Se-FA NPs). FA on the surface of Au@Ag_2_Se-FA is a pivotal targeting moiety. Given that FA has a high affinity for folate receptors, which are overexpressed on a wide range of cancer cells, this specific binding enables Au@Ag2Se-FA to be selectively internalized by tumor cells. Once inside, it could initiate a series of events that impact cellular immunity. For instance, it may enhance the recognition of tumor-associated antigens by T cells, promoting their activation and proliferation. This activation leads to a more robust and targeted attack on cancer cells. Moreover, The Ag ions released from the nanoparticle possess potent antibacterial and anticancer capabilities. These ions can disrupt the cellular structures and functions of both bacteria and cancer cells, effectively eliminating them.

Au@Ag_2_Se-FA restores cellular immunity in both anticancer and antibacterial contexts. By targeting cancer cells, releasing antigens, and promoting T-cell activation, it rejuvenates the antitumor immune response. Simultaneously, its antibacterial action helps maintain a healthy immune environment, enabling better-coordinated cellular immune responses against both tumors and bacteria, which ultimately contributes to improved patient outcomes and the potential for long-term relapse-free survival.

#### 5.4.4 Regulation of cell energy metabolism

Mitochondria are the key organelles for cellular energy metabolism. According to the endosymbiotic theory, mitochondria might originate from ancient bacteria. Due to the high dependence of tumor and bacterial cells on energy metabolism for maintaining the cell proliferation, targeted damage of mitochondria might have dual anticancer and antibacterial effects (Tehrani Nejad et al., 2022).

Hemiprotonic bisphenanthroline (ph-ph^+^) possesses practical antimicrobial and anticancer activities, partially through disrupting the energy metabolism of bacteria and tumor cells (Zhao et al., 2022a,b). Antibacterial experiments demonstrate that ph-ph^+^ has an excellent inhibitory effect on both Gram-positive and Gram-negative bacteria, exhibiting broad-spectrum antibacterial activity against *S. aureus, E. coli, S. pyogenes, S. pneumoniae, C. nucleatum, P. mirabilis, and B. fragilis*. In addition, ph-ph^+^ could induce apoptosis in tumor cells such as H22, B16, U251MG, and SH-SY5Y at the same concentration used for antibacterial activity. Although the anticancer and antibacterial mechanisms of ph-ph^+^ are not fully understood, preliminary studies suggest that they may be related to the inhibition of energy generation in eukaryotic cell mitochondria and bacteria.

#### 5.4.5 Others

Recently, thanks to the rapid advancement of chemical synthesis technology, emerging studies highlight novel strategies for dual-functional drug development. These innovative approaches leverage advances in synthetic biology and targeted delivery to address the complex tumor-microbe interplay. For instance, antibiotic-antibody conjugates (AACs) combine tumor-targeting antibodies with antimicrobial payloads, enabling selective delivery to infection-primed tumor microenvironments (Nurkesh et al., 2019). A study demonstrated that a ciprofloxacin-conjugated anti-EGFR antibody effectively eradicated Pseudomonas aeruginosa-infected colorectal tumors while sparing normal tissues. Small-molecule inhibitors such as quinoline-5-sulfonamides norfloxacin analogs, and zelkovamycin analogs possess anticancer and antibacterial activities (Zieba et al., 2024; Qurban et al., 2024). BET-HDAC dual inhibitors can prevent the proliferation of cancer cells and *C. albicans* (Huang et al., 2023). Moreover, polymersomes and carbon nanodots can function as drug carriers against cancers, fungi, and bacteria simultaneously (Nurkesh et al., 2019; Wang et al., 2022).

These compounds have diverse structures and can simultaneously combat tumors and potential infections, achieving a synergistic therapeutic effect. The synthesized compounds allow for precise chemical modifications to enhance potency and selectivity. They may act on multiple targets simultaneously or interfere with the physiological functions of cells through a completely new mechanism of action. This multi-target or new-mechanism mode of action increases the difficulty of drug resistance and is expected to delay or overcome the problem of drug resistance to a certain extent. Generally, these drugs exhibit strong antibacterial and anti-tumor activities. However, these results were obtained from experimental animals and need to be verified in clinical settings. Although these compounds possess antibacterial and anti-cancer activities, their molecular targets have not been fully elucidated. This means that we are not clear about which specific molecules the drugs interact with inside cells to exert their effects. This uncertainty makes it difficult to further optimize the drugs, predict drug responses, and understand potential side effects.

## 6 Conclusion and future perspectives

The intricate symbiosis between microorganisms and tumors presents both challenges and opportunities in modern oncology. Here we review the effects of microorganisms on cancer cell proliferation, infiltration, metastasis, drug resistance, and highlight the significance of single agents against both bacteria and cancer cells. The dual functional agents can simultaneously target tumor proliferation pathways and key active sites of pathogens, reducing clinical treatment complexity and minimizing the risk of multi-drug application. They also would achieve synergistic effects by decreasing the mutual promotion between pathogens and cancer cells.

Regarding the predictive biomarkers for the dual-functional therapies in patients, it may be considered to use the factors as predictive biomarkers of patient response to the therapies because bacterial infection and tumor infiltration are accompanied by the alteration in inflammation and immune factors (Zhang X. et al., 2024). In addition, 16S rRNA gene sequencing and genomic identification could achieve the comprehensive analysis of bacterial flora in tumor-bearing animals, which would be another method to evaluate the effects of the dual-functional therapies (Roje et al., 2024; Zeng et al., 2025).

Due to the persistent problem of concurrent infections in cancer patients, single agents with antibacterial and anticancer activities hold great promise. This innovative therapeutic strategy possesses transformative potential for oncology by employing a dual-targeting mechanism that simultaneously disrupts tumor-microbe symbiosis and modulates tumor-associated microbiota.
